# Supplementary material for: Enhanced Sensitivity of A549 Cells to Doxorubicin with WS2 and WSe2 Nanosheets via the Induction of Autophagy
Source: Int J Mol Sci. 2024 Jan 18;25(2):1164. doi: 10.3390/ijms25021164 (PMC10816038; doi:10.3390/ijms25021164)
Supplement: Supplementary file 1 [file ijms-25-01164-s001.zip › ijms-2752270-supplementary.pdf]

Supporting Information

Weitao Jin [1], Ting Yang [1], Jimei Jia [1], Jianbo Jia [2,*] and Xiaofei Zhou [1,3,*]

[1] College of Science & Technology, Hebei Agricultural University, Huanghua 061100, China; jin1631112@163.com (W.J.)
[2] Institute of Environmental Research at Greater Bay Area, Key Laboratory for Water Quality and Conservation of the Pearl River Delta, Ministry of Education, Guangzhou University, Guangzhou 510006, China
[3] Hebei Key Laboratory of Analysis and Control of Zoonotic Pathogenic Microorganism, Baoding 071000, China
* Correspondence: jb_jia@gzhu.edu.cn (J.J.); zhouxf@hebau.edu.cn (X.Z.)

Contents

Figure S1. Dose-dependent cytotoxicity of WS_2 or WSe_2 in A549 cells after incubation under different concentrations for 24 h.

Figure S2. Supernatant of WS_2 or WSe_2 did not contribute to autophagy induction.

Figure S3. Dose-dependent cytotoxicity of WS_2 or WSe_2 in U87 cells after incubation under different concentrations for 24 h.

Figure S4. WS_2 and WSe_2 sensitized U87 cells to DOX treatment.

Figure S5. Pre-treatment with WS_2 or WSe_2 induced more obvious cleavage of caspase 3 compared to cells treated with DOX.

Table S1. Zeta potentials of WS_2 and WSe_2.

Table S2. Fold up- or down-regulation of 84 autophagic genes by super array.

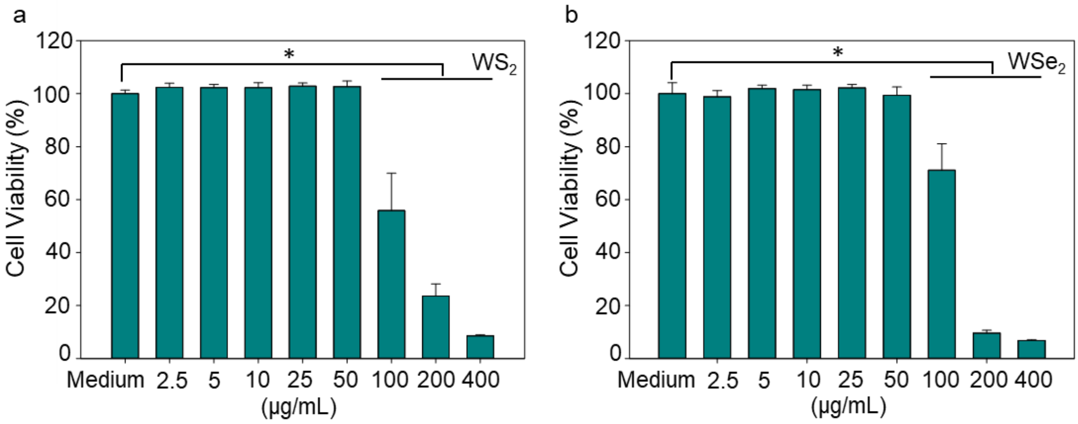

Figure S1. Dose-dependent cytotoxicity of WS_2 (a) or WSe_2 (b) in A549 cells after incubation under different concentrations for 24 h. Data were shown as mean ± s.d., n = 5. *P < 0.05.

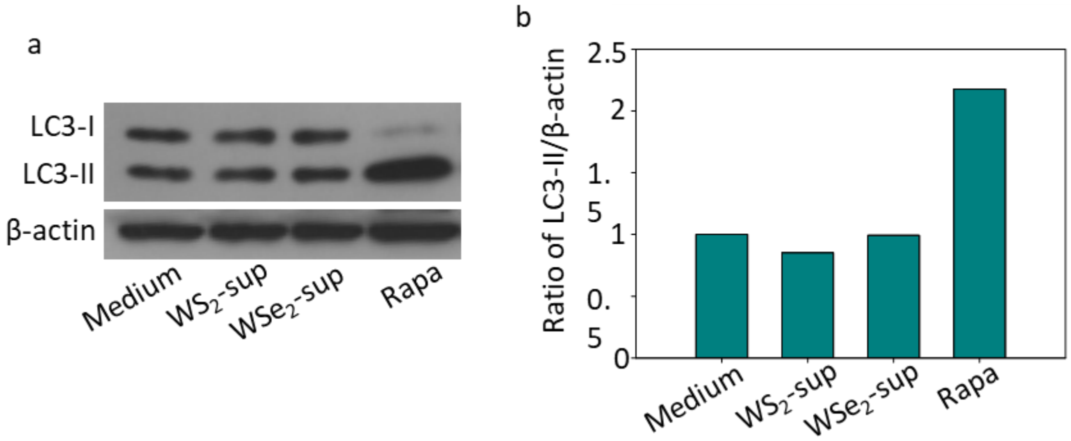

Figure S2. Supernatant of WS_2 or WSe_2 did not contribute to autophagy induction. (a) LC3-II formation in A549 cells after treatment with supernatant of WS_2 or WSe_2 as determined by Western blotting against LC3B antibody. A549 Cells treated with rapamycin (4 μM) for 12h was used as positive control. (b) LC3-II formation induced by the supernatant of WS_2 or WSe_2 was quantified by determining the ration of band intensities of LC3-II over β-actin using ImageJ.

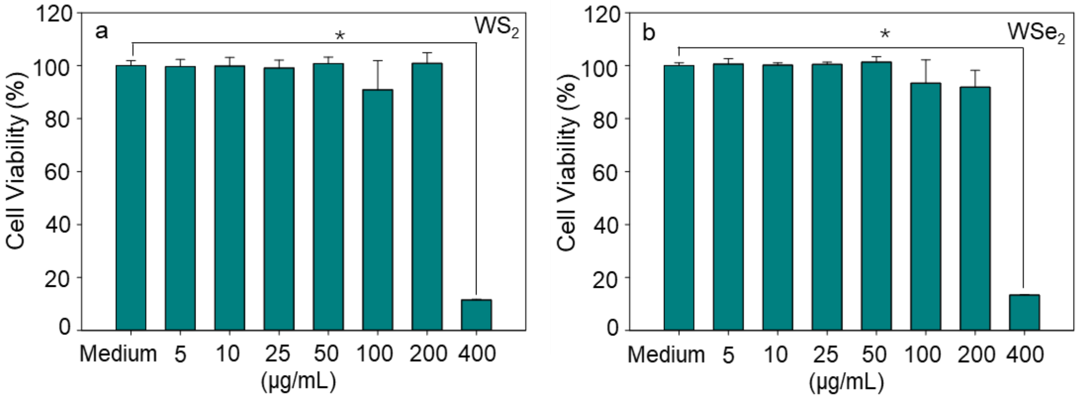

Figure S3. Dose-dependent cytotoxicity of WS_2 (a) or WSe_2 (b) in U87 cells after incubation under different concentrations for 24 h. Data were shown as mean ± s.d., n = 5. *P < 0.05.

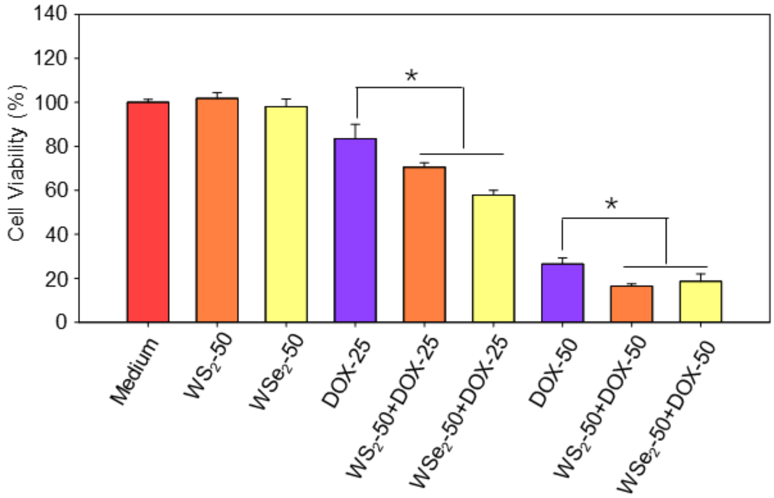

Figure S4. WS_2 and WSe_2 sensitized U87 cells to DOX treatment. (a) Pre-treatment with WS_2 or WSe_2 nanosheets enhanced the DOX-induced toxicity in U87 cells. Data are shown as the mean ± s.d.; n = 5; *P < 0.05.

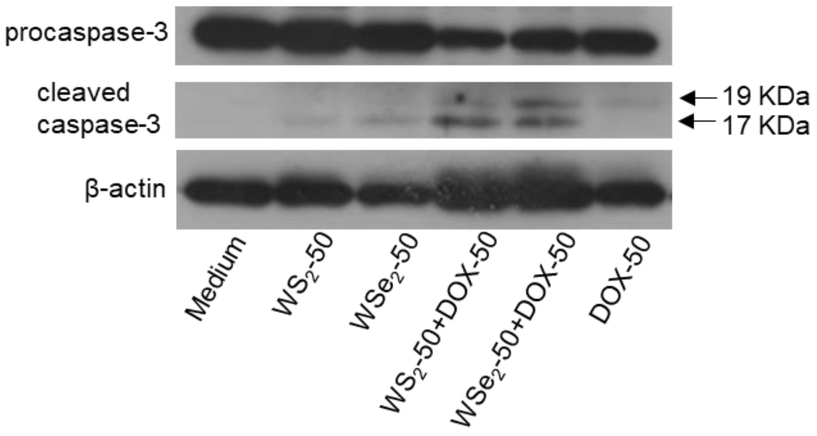

Figure S5. Pre-treatment with WS_2 or WSe_2 induced more obvious cleavage of caspase 3 compared to cells treated with DOX.

Table S1. Zeta potentials of WS_2 and WSe_2.

	WS_2	WSe_2
Zeta potential in water (mV)	-39.0 ± 1.9	-41.2 ± 1.5
Zeta potential in 10%FBS (mV)	-11.3 ± 1.2	-11.7 ± 0.9

Table S2. Fold up- or down-regulation of 84 autophagic genes by super array.

Genes	WS_2/control	WSe_2/control
AKT1	0.74	0.60
AMBRA1	0.76	0.64
APP	0.50	0.32
ATG10	0.57	0.48
ATG12	0.69	0.57
ATG16L1	0.66	0.75
ATG16L2	1.34	0.81
ATG3	0.82	0.66
ATG4A	0.53	0.38
ATG4B	1.06	0.77
ATG4C	0.64	0.49
ATG4D	0.62	0.43

ATG5	0.70	0.49
ATG7	0.80	0.60
ATG9A	0.72	0.54
ATG9B	2.06	0.81
BAD	0.50	0.28
BAK1	0.68	0.58
BAX	0.65	0.55
BCL2	1.75	2.34
BCL2L1	0.61	1.18
BECN1	0.80	0.55
BID	0.55	0.58
BNIP3	0.58	0.55
CASP3	0.65	0.59
CASP8	0.73	0.52
CDKN1B	0.69	0.51
CDKN2A	0.81	0.81
CLN3	0.59	0.35
CTSB	0.78	0.34
CTSD	0.93	0.59
CTSS	1.01	0.71
CXCR4	0.81	0.81
DAPK1	3.05	1.52
DRAM1	0.45	0.44
DRAM2	0.52	0.28
EIF2AK3	0.75	0.46
EIF4G1	0.81	0.74
ESR1	0.92	0.31
FADD	0.56	0.55
FAS	0.52	0.67
GAA	0.85	1.07
GABARAP	0.67	0.41
GABARAPL1	0.77	0.22
GABARAPL2	0.63	0.48
HDAC1	0.66	0.48
HDAC6	1.14	0.59
HGS	5.91	6.47
HSP90AA1	0.44	0.48
HSPA8	0.47	0.62
HTT	0.89	0.63
IFNG	0.81	0.81
IGF1	1.77	0.81
INS	0.81	0.81
IRGM	0.81	0.81

LAMP1	0.80	0.59
MAP1LC3A	0.22	0.10
MAP1LC3B	0.93	0.63
MAPK14	0.64	0.42
MAPK8	0.58	0.43
MTOR	0.79	0.60
NFKB1	1.36	1.39
NPC1	0.97	0.74
PIK3C3	0.59	0.46
PIK3CG	0.81	0.81
PIK3R4	0.70	0.71
PRKAA1	0.68	0.59
PTEN	0.96	0.57
RAB24	1.00	0.91
RB1	0.71	0.50
RGS19	1.01	1.06
RPS6KB1	0.70	0.53
SNCA	0.33	0.15
SQSTM1	0.97	0.46
TGFB1	0.77	0.76
TGM2	0.71	1.01
TMEM74	1.00	0.39
TNF	3.06	0.81
TNFSF10	0.85	0.06
TP53	1.01	0.72
ULK1	2.55	1.37
ULK2	0.81	0.60
UVRAG	0.70	0.58
WIPI1	0.91	0.69
ACTB	1.00	1.23
B2M	0.68	0.61
GAPDH	1.39	1.44
HPRT1	0.59	0.60
RPLP0	0.72	0.57
